# Annual acknowledgement of manuscript reviewers

**DOI:** 10.1186/1746-4358-9-3

**Published:** 2014-02-17

**Authors:** Lisa Amir

**Affiliations:** 1 La Trobe University, Melbourne, Australia; 2 University of Melbourne, Melbourne, Australia

## Abstract

**Contributing reviewers:**

The editor of *International Breastfeeding Journal* would like to thank all our reviewers who have contributed to the journal in Volume 8 (2013).

## 

Clara Aarts

Sweden

Sheryl Abrahams

United States of America

Kingsley Agho

Australia

Alex Anderson

United States of America

Genevieve Becker

Ireland

Nina Berry

Australia

Colin Binns

Australia

Wendy Brodribb

Australia

Adrian Cameron

Australia

Katherine Carroll

Australia

Adriano Cattaneo

Italy

Ilana Chertok

United States of America

Ellen Chetwynd

United States of America

Yoo-Mi Chung

South Korea

Anna Coutsoudis

South Africa

Meabh Cullinane

Australia

Bithika Das

Australia

Mary-Ann Davey

Australia

Susan Donath

Australia

Gudina Egata

Ethiopia

Abdel-Hady El-Gilany

Egypt

Megan Elliott-Rudder

Australia

Ingunn Engebretsen

Norway

Catherine Fetherston

Australia

Della Forster

Australia

Betsy Foxman

United States of America

Suzanne Garland

Australia

Ted Greiner

South Korea

Debra Hector

Australia

Pamela Hill

United States of America

Jenny Ingram

United Kingdom

Helene Johns

Australia

Sue Jordan

United Kingdom

Linda Kvist

Sweden

Kenneth Maleta

Malawi

Susana Matias

United States of America

Karalyn McDonald

Australia

Helen McLachlan

Australia

Melina Mgongo

Tanzania

Karen Marie Moland

Norway

Judith Myers

Australia

Antonia Nelson

United States of America

Nathan Nickel

Canada

Joy Noel-Weiss

Canada

Funmilola OlaOlorun

Nigeria

Antonio Oliver-Roig

Spain

Gillian Opie

Australia

Luiz Prestes-Carneiro

Brazil

Nikki Rogers

United States of America

Nigel Rollins

South Africa

Heather Rowe

Australia

Kath Ryan

Australia

Magda Sachs

United Kingdom

Moni Saha

Australia

Virginia Schmied

Australia

Jane Scott

Australia

Sonia Semenic

Canada

Touran Shafiei

Australia

Karen Simmer

Australia

Rhonda Small

Australia

Natasha Sriraman

United States of America

David Tappin

United Kingdom

Charlotte Tawiah-Agyemang

Ghana

Thorkild Tylleskar

Norway

Ravi Upadhyay

India

Lisa Vallely

Papua New Guinea

John Vince

Papua New Guinea

Louise Wallace

United Kingdom

Jane Yelland

Australia

